# Task-shifting through community health workers: factors influencing access and utilization of modern family planning methods in Ziniaré, Burkina Faso

**DOI:** 10.3389/frph.2026.1650177

**Published:** 2026-02-17

**Authors:** Fatimata Koinda, Tabither Gitau, Wilfred Zoungrana, Erick Yegon, Nzomo Mwita, Assetou Zongo, Lamissi Sawadogo, Alice Koimur, Claire Nyabonyi, Rhonnie Omondi Omollo, Joseph Kyalo Njoroge, Andre Ky Yolland, Mahamadi Tassembedo

**Affiliations:** 1 Programs Department, Living Goods, Ouagadougou, Burkina Faso; 2 Programs Department, Living Goods, Nairobi, Kenya; 3 Directorate of Community Health, Ministry of Health, Ouagadougou, Burkina Faso

**Keywords:** community health workers (Agents de santé à Base communautaire – ASBCs), contraceptive use, education, modern family planning, multivariable logistic regression, sociocultural barriers

## Abstract

**Introduction:**

Burkina Faso has expanded access to family planning (FP) with modern contraceptive use prevalence among married women reaching 32% in 2021. However, regional disparities persist, especially in rural Ziniaré district where unmet needs among married women remain high (20%). Barriers like stigma, limited youth-friendly services, and low contraceptive awareness hinder uptake. This study examined factors influencing modern FP use and explored the role of community health workers, *Agents de Santé à Base Communautaire* (ASBCs), a government-supported cadre trained and equipped to provide FP services at community level, within a task-shifting intervention. Understanding these factors is vital for strategies to expand equitable access and improve reproductive health in rural and peri-urban areas.

**Methods:**

We conducted a cross-sectional comparative mixed-methods implementation study with quasi-experimental features, following TREND reporting guidelines, in Ziniaré district, in August 2024. The study included four intervention and three control communes. Intervention communes received an eight-month package for ASBCs that included FP training, provision of short-acting contraceptives (oral pills, subcutaneous DMPA, condoms), regular supportive supervision and structured performance monitoring. Control communes continued to provide routine, primarily facility-based FP services. Quantitative data were collected from 282 women aged 15–49 years (136 intervention, 146 control) using structured household questionnaires; analyses included descriptive statistics and multilevel logistic regression (*p* < 0.05). Qualitative data were obtained from 60 participants (men, ASBCs, facility managers) through semi-structured interviews.

**Results:**

Modern contraceptive prevalence was 48% in the intervention group and 53% in the control group, without statistical significance (OR = 0.68, 95% CI: 0.27–1.68, *p* = 0.4). Factors associated with higher contraceptive use included being in a union (OR = 21.3, *p* = 0.009), knowing more contraceptive methods (OR = 1.45, *p* < 0.001), and discussing FP with a partner (OR = 7.57, *p* < 0.001). Most women preferred obtaining FP services at health facilities for confidentiality (82% vs. 17% for community-based). Qualitative findings highlighted persistent sociocultural and religious barriers, including stigma and myths about sterility.

**Conclusion:**

Despite ASBC-led task-shifting improving awareness, engagement, and access to FP services, the difference in contraceptive prevalence between intervention and control communes (48% vs. 53%, OR = 0.68, 95% CI: 0.27–1.68, *p* = 0.4) does not provide strong evidence of a measurable impact. The study was likely underpowered to detect this within the limited 8-month period. Strengthening community-based FP requires longer implementation, enhanced confidentiality, culturally sensitive strategies, education promotion, male engagement and continued ASBC support.

## Introduction

Family planning (FP) is an essential strategic component of public health, particularly in low- and middle-income countries (LMICs). It is a critical intervention for reducing maternal and child morbidity and mortality, preventing unintended pregnancies, and advancing the sexual and reproductive rights of women and girls (1, 2). Access to high-quality FP services is fundamental not only to reproductive autonomy but also to broader societal goals of gender equality, poverty reduction, and sustainable development. Universal provision of modern contraception can reduce maternal mortality by an estimated 44%, prevent millions of unsafe abortions annually, and support women's education and economic empowerment (3–5).

Globally, contraceptive use among women aged 15–49 rose from 33.2% in 1970 to 51.9% in 2019 (6). Nevertheless, unmet needs for contraception remain substantial in 2019, 8.3% of women wished to avoid pregnancy but lacked access to a method (6). These inequalities are particularly pronounced in LMICs (low- and middle-income countries), where barriers include geographical isolation, restrictive sociocultural norms, supply chain disruptions, and shortages of skilled health personnel (7). In response, global initiatives such as the Millennium Development Goals (MDGs), the Sustainable Development Goals (SDGs), and Family Planning 2020 (FP2020) and its successor FP2030 have mobilized political commitment, financial investment, and innovation to expand contraceptive access, particularly in sub-Saharan Africa (8).

In Burkina Faso, FP has been on the public health agenda since the 1990s, following the country's commitment to the International Conference on Population and Development (ICPD) in 1994. The government progressively integrated FP into maternal and child health programs, supported by international partners through awareness campaigns, service delivery initiatives, and the development of a national FP strategy in the early 2000s. These efforts contributed to gradual improvements in contraceptive uptake, with the prevalence of modern contraceptive use among married women increased from 15% in 2010 to 32% in 2021 (9). The country has pursued multiple approaches, including national campaigns, community outreach, and partnerships with international agencies such as UNFPA and FP2030. To increase access in underserved communities, the Ministry of Health piloted a task-shifting policy in 2014, authorizing community health workers (Agents de Santé à Base Communautaire, ASBCs) to deliver selected FP services in a limited number of districts. Over time, the initiative has expanded gradually but remains limited: not all districts are covered, and many ASBCs have yet to be trained, supplied with contraceptive products, or supported with structured supervision for FP delivery.

Community health workers (Agents de Santé à Base Communautaire, ASBCs) are selected by their village and trained in basic primary health care, promotional and preventive services but typically have shorter and more focused training compared to health facility staff. Their role is primarily to deliver community-based services, provide health education, and refer clients to health facilities when needed. Data from the Performance Monitoring for Action (PMA) Burkina Faso survey highlight the growing role of ASBCs as sources of FP information, particularly among young women: 13% of those aged 15–19, 29% aged 20–24, and 29% aged 25–49 reported receiving FP information from community health workers (10).

Despite national improvements in modern contraceptive use, significant gaps in access and utilization remain, particularly in rural and underserved communities. Regional disparities are notable, especially in Ziniaré district, where unmet need among married women remains high (20%). Although the Ministry of Health has piloted a task-shifting policy for ASBCs to deliver selected FP services, its implementation is limited in scope, and many ASBCs have yet to be trained, supplied with contraceptive products, or integrated into supportive supervision systems. Consequently, communities continue to face barriers in accessing high-quality FP services, including geographic isolation, limited knowledge, and socio-cultural constraints.

In this context, Living Goods’ DESC initiative in Ziniaré provides an opportunity to strengthen ASBC capacity and assess how integrated digital tools, commodities, supervision, and incentives influence the adoption and use of modern contraceptives. Understanding community knowledge, perceptions, and service delivery challenges is critical to inform policy and optimize task-shifting strategies in similar settings.

Since 2023, Living Goods has been supporting the Ministry of Health in the learning site of Ziniaré district to strengthen community health through the DESC model (Digitally enabled, Equipped, Supervised, and Compensated). Within this framework, ASBCs receive smartphones loaded with the smart health app (eSanteCoM application), regular supplies of essential health commodities, supportive supervision, and financial incentives. In 2024, Living Goods expanded this support to family planning by training ASBCs on FP modules, supplying them with short-acting contraceptive methods [oral pills, subcutaneous DMPA (formerly known as Sayana Press), condoms], and extending the existing supervision system to include FP service delivery. This complementary initiative enhances the Ministry's task-shifting policy while embedding FP services into a broader primary health care strategy.

In this study, we evaluated the task-shifting policy for FP delivery among ASBCs as implemented and enhanced through the DESC initiative. Specifically, it explores community knowledge and perceptions of FP, male involvement, challenges faced by ASBCs and facility managers, and factors influencing the adoption and use of modern contraceptive methods.

## Methodology

### Study design

We conducted a cross-sectional comparative mixed-methods study with quasi-experimental features following TREND reporting guidelines, in Ziniaré district, Burkina Faso, in August 2024. The study sites were selected based on the operational implementation strategy of the Living Goods DESC initiative in collaboration with the Ministry of Health. The design compared non-randomly selected intervention communes where task-shifting of family planning (FP) services to community health workers, Agents de Santé à Base Communautaire (ASBCs), was actively implemented and supported, with control communes where FP services remained primarily health facility-based (Centres de Santé et de Promotion Sociale, CSPS). While ASBCs in both arms provided FP counseling and received supervision for routine community health activities, in intervention communes they were additionally trained and authorized to dispense short-acting contraceptives (oral pills, subcutaneous DMPA, condoms) within the community, receive supportive supervision explicitly extended to FP, and participate in systematic performance monitoring. In this study, task-shifting refers specifically to the formal delegation of FP service delivery to ASBCs, including counseling and provision of short-term contraceptive methods, whereas supportive supervision and coaching are considered complementary mechanisms to ensure quality and sustainability. Quantitative and qualitative data were collected in parallel during the same period across both intervention and control communes. The design allows for the assessment of factors associated with family planning uptake, as well as the implementation of task-shifting family planning services to *Agents de Santé de Base Communautaire* (ASBCs) in Ziniaré District, while recognizing that causal attribution is limited due to its quasi-experimental cross-sectional nature. This study was implemented within the framework of Burkina Faso's national FP policy, which supports task-shifting of contraceptive services to ASBCs.

### Intervention

In the intervention group (Nagréongo, Loumbila, Dapélogo, and Ourgou Manéga), ASBCs were supported under the Living Goods DESC model (Digitally enabled, Equipped, Supervised, and Compensated) and received additional FP-specific inputs over an eight-month period (January–August 2024). These inputs included the provision of family planning commodities, ensuring a regular supply of oral contraceptive pills, condoms, and subcutaneous depot medroxyprogesterone acetate (DMPA-SC). ASBCs also benefited from supportive supervision and coaching, with structured mentoring on FP counseling, community engagement, and client follow-up. Supervisors conducted monthly field visits that combined direct observation, feedback, and problem-solving sessions. In addition, ASBCs were engaged in structured performance monitoring specifically for FP, where monthly targets were set, progress was regularly reviewed, and feedback was systematically provided through supervision sessions. Monthly performance targets for FP service delivery were supported by data review sessions (feedback loops) between ASBCs and supervisors to enhance accountability and facilitate problem-solving. Implementation fidelity of the family planning intervention across communes, including ASBC density, supervision frequency, stockout occurrence, intervention start dates, and attainment of performance targets, is summarized in Table 1.

**Table 1:** Family planning intervention fidelity by commune: ASBC density, supervision cadence, stockouts, start dates, and performance targets attained.

Commune	Number of ASBCs	Women of reproductive age (WRA) per ASBC	Supervision cadence (month)	% of ASBC with stockouts	Start date of FP-specific activities	New users for pills and injectables:^a^ Average Performance targets attained (%)
Dapelogo	24	237	1	0.30%	Jan-24	55.70%
Nagreongo	10	181	1	0.90%	Jan-24	61.50%
Ourgou Manega	22	117	1	3.90%	Jan-24	137.50%
Loumbila	30	179	1	0.60%	Jan-24	83.60%

WRAs not currently using (never used + used but stopped) initiated on FP

**Figure 1:**
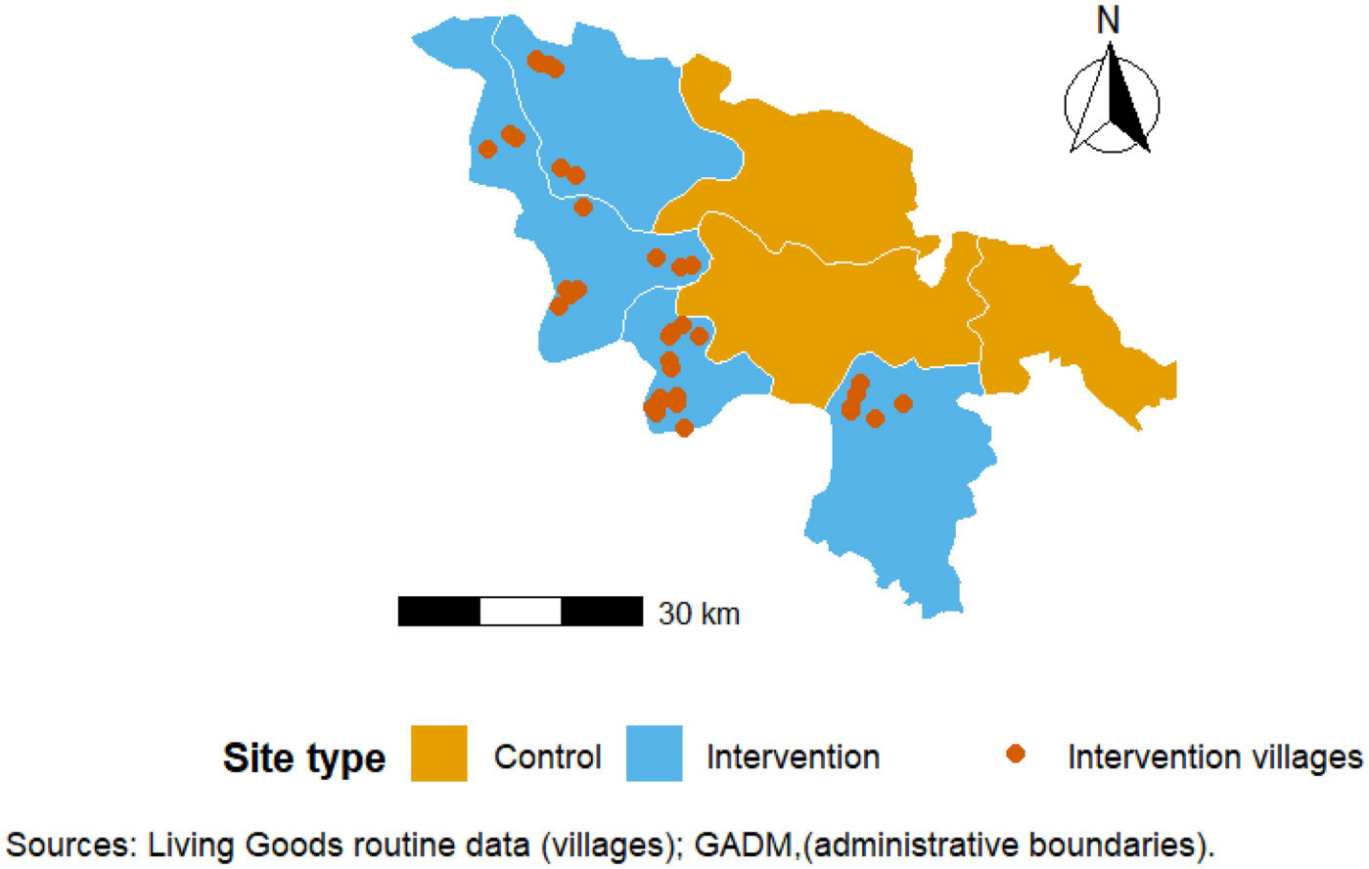
Geographic distribution of intervention and control sites in Ziniaré district, Burkina Faso.

In the control communes (Absouya, Zitenga, and Ziniaré town), ASBCs were part of DESC for general health services but did not receive FP-specific commodities, supervision, or structured FP performance monitoring. Family planning services in these areas remained primarily facility-based, without targeted community-level performance management for FP. The geographic distribution of intervention and control communes within Ziniaré District is presented in Figure 1.

### Study population and sampling

The quantitative survey targeted women of reproductive age (15–49 years) residing in intervention and control communes*.* A village-based sampling strategy was employed, beginning with a household listing to identify eligible women in each village. From this list, ten households per village were randomly selected, covering a total of 27 villages, 13 in the intervention group and 14 in the control group. In addition, purposive considerations were applied to ensure socio-demographic diversity, particularly with respect to age, marital status, and education.

### Sample size determination

Based on *a priori* power calculations (*α* = 0.05, 80% power), a minimum of 252 women was required to detect a meaningful difference in FP use between groups, assuming a baseline prevalence of 32%, a detectable difference of 18%, and an intra-cluster correlation coefficient (ICC) of 0.02. These assumptions and corresponding detectable effect parameters are summarized in Appendix Table A1.

A total of 282 women were enrolled and interviewed (136 intervention, 146 control), exceeding the required minimum. Cluster sizes ranged from 1 to 20 participants (mean = 9.10, SD = 3.53, CV = 0.39). *post-hoc* detectable effect calculations were performed using these achieved sample sizes and observed cluster sizes, with ICC = 0.02, applying the standard design-effect–adjusted formula for two-sample comparisons of proportions. Appendix Table A1 presents the full *post-hoc* calculations, and Appendix Table A2 provides a sensitivity analysis for ICC values ranging from 0.01 to 0.03. The resulting minimum detectable differences substantially exceed the observed 5-percentage-point difference (48% vs. 53%), confirming that the study was underpowered to detect this small effect.

The qualitative component purposively sampled men, ASBCs, and health facility managers to explore FP barriers, male involvement, and implementation experiences. Participants were selected to maximize variation in age, sex, and roles, and data collection continued until thematic saturation was reached, that is, when no substantially new insights emerged. Although women were not directly included in the qualitative interviews, a limitation acknowledged in this study, the survey data served as an important supplementary source, providing valuable insights into women's perspectives.

### Baseline characteristics and non-random commune selection

As commune-level pre-intervention data on modern contraceptive prevalence and women's education were not available (EDS 2021 provides only district- or regional-level estimates), ASBC system capacity was the only comparable indicator accessible across all communes. Appendix Table A3 presents pre-intervention data on the total number of ASBCs and the ratio of Women of Reproductive Age (WRA) per ASBC for each commune. These figures serve as proxies for baseline ASBC coverage. Baseline ASBC coverage was broadly comparable between intervention and control communes (mean WRA/ASBC: 171 vs. 171, respectively), suggesting no major imbalance in community health system capacity prior to implementation.

### Causual framework

**Figure 2:**
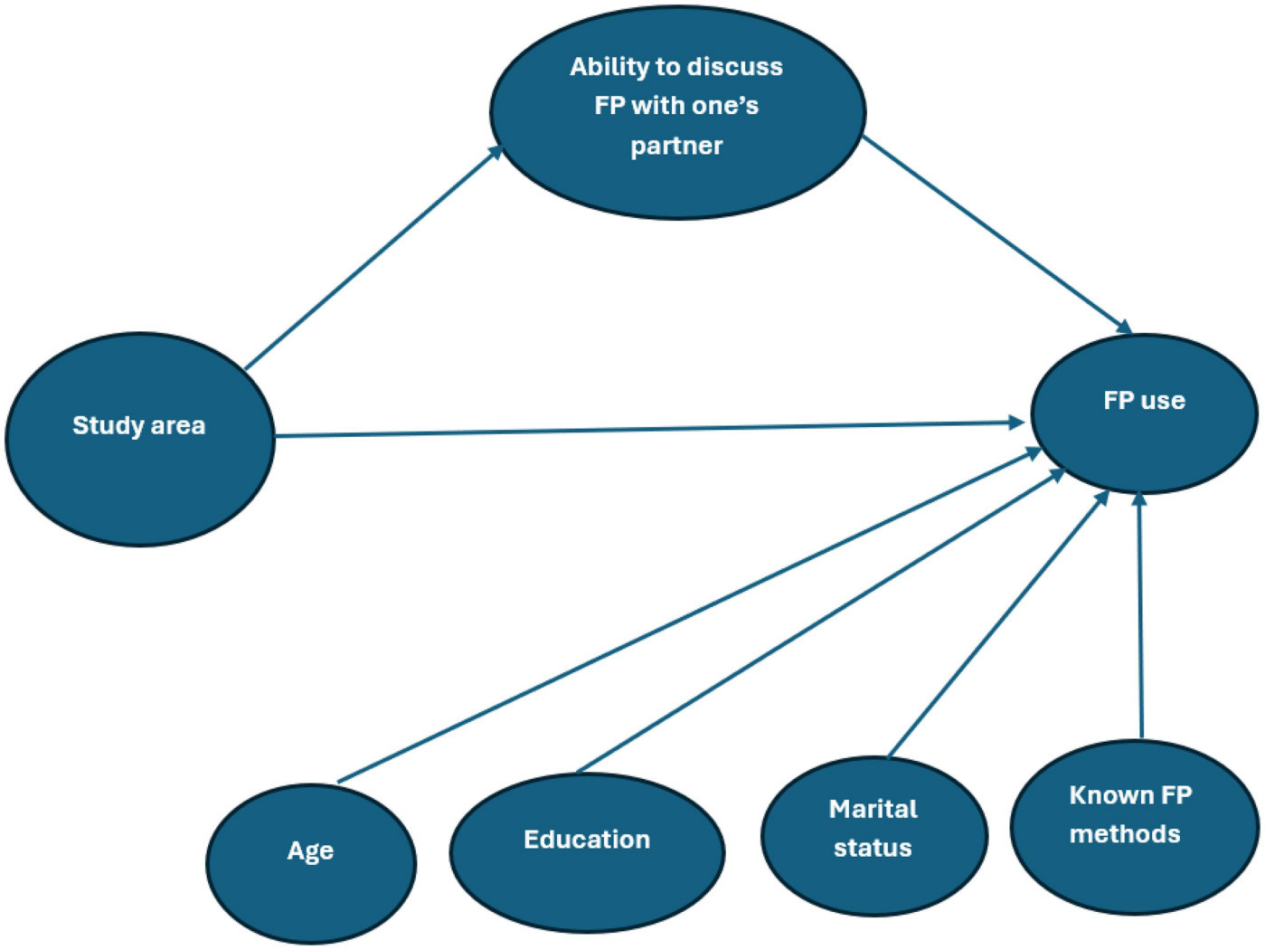
Directed acyclic graph (DAG) illustrating hypothesized causal pathways between task-shifting intervention, socio-demographic factors, and family planning outcomes.

To guide our quantitative analyses and clarify assumptions regarding potential confounding, we constructed a Directed Acyclic Graph (DAG) illustrating the hypothesized relationships between intervention exposure, socio-demographic factors, and family planning outcomes (Figure 2). The DAG depicts the pathways through which the task-shifting intervention, individual characteristics (e.g., age, education, marital status), and contextual factors (e.g., commune-level implementation differences) may influence both FP knowledge and modern FP use.

Importantly, **partner communication about FP was modeled as a mediator rather than a confounder**. This decision reflects our theoretical assumption that the intervention may enhance partner communication, through increased knowledge, empowerment, or counseling effects, which in turn can influence contraceptive uptake. In the DAG, partner communication lies on the causal pathway between exposure to the intervention and FP outcomes, rather than preceding the intervention.

Modeling it as a confounder would imply that partner communication independently affects both exposure and outcome (i.e., that it predates exposure), which is inconsistent with the program's logic model and implementation timeline. Aligning with the DAG, we therefore treated partner communication as a **mechanistic pathway** linking the intervention to behavioral outcomes, rather than a source of confounding bias.

### Data collection

The quantitative component consisted of a structured household questionnaire administered to women of reproductive age (15–49 years). The tool captured socio-demographic characteristics, knowledge and use of family planning (FP) methods, partner communication about FP, exposure to FP messages, and access to FP services. Questionnaires were pre-tested, locally adapted, and administered in August 2024 to ensure temporal alignment between intervention and control areas.

The qualitative component included key informant interviews (KIIs) with facility managers (*n* = 7) and focus group discussions (FGDs) with community health workers (ASBCs, *n* = 18) and male community members (*n* = 7). Women were not directly included in the qualitative interviews. While survey data provided insights into women's perspectives, this exclusion may bias qualitative interpretations toward provider and male perspectives.

### Outcome definitions

The primary outcomes of interest were the current use of modern family planning (FP) methods and knowledge of FP methods. Modern FP use was defined as the current use of at least one modern contraceptive method, including sterilization, implants, intrauterine devices (IUDs), injectables, pills, or condoms. Women reporting the use of multiple methods were classified as users for the binary outcome variable (yes/no). Knowledge of FP methods was assessed spontaneously by asking women to name all modern contraceptive methods they knew without prompts, and the outcome was operationalized as the total number of modern methods correctly identified by each participant.

### Data analysis

#### Quantitative analysis

Quantitative data were summarized using descriptive statistics (frequencies, percentages, and means) to characterize family planning (FP) knowledge, use, perceptions, and access.

For inferential analyses, multilevel mixed-effects models were applied to identify factors associated with FP knowledge and contraceptive uptake, accounting for clustering. Potential baseline differences between intervention and control communes were statistically accounted for using these models.

Two outcomes were analyzed. The number of modern contraceptive methods known (count outcome) was modeled using a COM-Poisson mixed model (glmmTMB) with a log link, including random intercepts for communes and villages. Modern contraceptive use (binary outcome) was analyzed using a logistic mixed-effects model (lme4) with a logit link and a random intercept for villages. Random slopes were tested but not included, as preliminary exploration indicated they were unnecessary.

Covariates (age, education, marital status and contraceptive knowledge) were selected based on a Directed Acyclic Graph (DAG) to rigorously control potential confounding inherent to the quasi-experimental design. Missing data were minimal (*n* = 2) and were removed through complete-case analysis.

Model diagnostics, including assessment of dispersion, Pearson *χ*^2^/df, and goodness-of-fit, were conducted. Detailed results, including intra-class correlations (ICCs), design effects, and model performance indices, are reported in Appendix Table A4.

Chi-square analyses were adjusted for multiple comparisons using the Benjamini–Hochberg false discovery rate (FDR) method to control the Type I error rate, which is appropriate for exploratory analyses. All analyses were conducted in R (see Appendix A2 for software details, package versions). Results are reported as adjusted odds ratios or rate ratios with 95% confidence intervals.

#### Qualitative analysis

Transcripts from key informant interviews (KIIs) and focus group discussions (FGDs) were analyzed using inductive thematic analysis. An initial codebook was developed based on a subset of transcripts independently coded by two researchers, with discrepancies resolved through discussion to ensure coding reliability. Triangulation of data sources and methods, including KIIs with facility managers, FGDs with ASBCs, and FGDs with male community members, was performed to enhance the validity and depth of interpretation. Data collection continued until thematic saturation was reached, defined as the point at which no new substantial insights emerged. Qualitative findings were subsequently triangulated with quantitative results to enrich the understanding of key determinants of contraceptive use, partner communication, and service delivery preferences.

### Ethical considerations

Ethical approval was obtained from the National Ethics Committee for Health Research (CERS), Ministry of Health of Burkina Faso (Ref. No. 2024-07-211). Written informed consent was obtained from all participants; illiterate participants were assisted by a witness of their own choosing. For adolescents aged 15–17 years, parental or guardian consent was obtained alongside the adolescent's written assent using age-appropriate language. To safeguard privacy, particularly for sensitive topics such as reproductive health and family planning, interviews were conducted in private settings without parental presence. All responses were anonymized, confidentiality was strictly maintained, and data were securely stored with access limited to the research team.

## Results

The study enrolled 282 women of reproductive age. The overall modern contraceptive prevalence across all communes was 50% (*n* = 142/282). A comparison between the two study arms showed that in the intervention group, the prevalence was 48% (*n* = 65/136), slightly lower than the 53% (*n* = 77/146) observed in the control group. The difference between the two groups was not statistically significant (OR = 0.68, 95% CI: 0.27–1.68 *p* = 0.4).

### Socio-demographic characteristics of study participants

The study participants exhibit several key socio-demographic characteristics that influence their FP utilization. As shown in Table 2, slightly over half (53.2%), of the women surveyed are illiterate, with a slightly higher rate (60.3%) in the control group compared to the intervention group (45.6%). Overall, 94.7% of the women are married or cohabiting with no statistically significant difference in marital status between the groups (*p*-value = 0.9). The average age of the participants is 27.5 years, indicating a relatively young demographic with specific reproductive health needs. These demographic factors collectively shape the context within which FP services are accessed and utilized.

**Table 2:** Socio-demographic characteristics of study participants by group.

Variables	Control *n* = 146^a^	Treatment *n* = 136^a^	Overall *n* = 282^a^	*p*-value
Education				0.046 (Chi^2^)
Illiterate	88 (60.3%)	62 (45.6%)	150 (53.2%)	
Primary	34 (23.3%)	42 (30.9%)	76 (26.9%)	
Secondary +	24 (16.4%)	32 (23.5%)	56 (19.9%)	
Marital status				0.9 (Chi^2^)
Single	7 (4.8%)	8 (5.9%)	15 (5.3%)	
Married/co-habiting	139 (95.2%)	128 (94.1%)	267 (94.7%)	
Age group				0.004 (Chi^2^)
[16–20]	27 (18.5%)	10 (7.4%)	37 (13.1%)	
[21–35]	110 (75.3%)	107 (78.7%)	217 (76.9%)	
[36–47]	9 (6.2%)	19 (13.9%)	28 (10.0%)	
Age mean (SD)	27 (6.0)	28 (6.5)	27.5 (6.2)	0.172(*t*-test)

a *n* (%).

### Contraceptive methods known

Analysis of contraceptive methods known reveals that women in the control group are familiar with a slightly higher average of 5.8 methods compared to 5.2 methods in the intervention group. This difference was statistically significant (*p*-value = 0.0046). This suggests that awareness of available contraceptive methods was broadly high but somewhat more diversified in the control sites (Table 3).

**Table 3:** Contraceptive methods known by study group.

Variables	Overall *n* = 282	Control *n* = 146	Treatment *n* = 136	*p*-value
Number of Contraceptive methods known				0.0046 (*t*-test)
Mean (SD)	5.5 (1.8)	5.8 (1.6)	5.2 (1.8)	
Median	6.0	6.0	5.0	
Min–Max	0.0–8.0	0.0–8.0	0.0–8.0	

Interestingly, men's knowledge of contraceptive methods is reported as quite limited, as illustrated by participant quotes:

“I don't know any but what I know is that for women there is what is suitable.” FGD-men

“I have no idea about the different types. It’s when women go to the hospital and get the type that suits them, that’s what’s good.” FGD-men

Regression analysis identified several factors influencing the number of contraceptive methods known. Participation in gatherings for educational talks and events was significantly associated with higher knowledge: women who attended such gatherings reported knowing 17% more methods than those who did not (IRR = 1.17, 95% CI:1.08–1.27, *p* < 0.001). Educational attainment also appeared to play an important role: compared to women with secondary or higher education (reference category), those who were illiterate or had only primary schooling reported knowing 12% and 10% fewer methods, respectively (IRR = 0.88, 95% CI: 0.79, 0.97; and IRR = 0.90, 95% CI: 0.82, 0.99) (Table 4).

**Table 4:** Generalized linear mixed model (COM-poisson) results for factors associated with the number of contraceptive methods known.

Variables	IRR	95% CI^a^	*p*-value
Study area			
Control	—	—	
Treatment	0.921	0.72, 1.18	0.51
Gathering			
No	—	—	
Yes	1.17	1.08, 1.27	<0.001
Age	1.0	0.99, 1.01	0.21
Marital status			
Single	—	—	
Married/co-habiting	1.02	0.86, 1.21	0.83
Education			
Secondary+	—	—	
Primary	0.90	0.82, 0.99	0.045
Illiterate	0.88	0.79, 0.97	0.009

a CI, confidence interval.

Cluster-level variation was low (ICC_commune = 0.02; ICC_village = 0.025), indicating that only 2%–2.5% of the variance in contraceptive knowledge was attributable to differences between communes or villages. However, the design effects were non-negligible (DEFF_commune = 1.77; DEFF_village = 1.20), reflecting inflation of the standard errors due to clustering. The full variance decomposition, including ICC and design effect estimates, is provided in Appendix Table A4. These results justify the use of multilevel modeling to obtain valid standard errors and confidence intervals.

These findings indicate that participation in community gatherings and higher educational attainment are independently associated with greater contraceptive knowledge, after accounting for clustering and individual-level confounding.

### Sources of information on modern FP

**Analysis of the primary sources of information on family planning (FP)** revealed that **community-based communication channels** were the dominant sources of information among women. As shown in Table 5, **social gatherings** were the most frequently cited source overall, reported by **59%** of respondents (67% in the control group vs. 49% in the treatment group, *p* *=* *0.008*). This highlights the central role of interpersonal and community networks in the dissemination of FP information within the study population.

**Table 5:** Sources of information on family planning among women by study group.

Sources of information on FP	Control *n* = 146^a^	Treatment *n* = 136^a^	Overall *n* = 282^a^	*p*-value
Radio	66 (45%)	72 (53%)	138 (49%)	0.3 (Chi^2^)
Television	22 (15%)	14 (10%)	36 (13%)	0.3 (Chi^2^)
Newspaper/magazine	17 (12%)	7 (5.1%)	24 (8.5%)	0.12 (Chi^2^)
Phone/sms	22 (15%)	7 (5.1%)	29 (10%)	0.023 (Chi^2^)
Social gatherings	98 (67%)	67 (49%)	165 (59%)	0.008 (Chi^2^)

a *n* (%).

**Radio** ranked as the second most common source, mentioned by **49%** of women (45% in the control group vs. 53% in the treatment group, *p* *=* *0.3*), confirming its importance as a widely accessible traditional medium.

In contrast, modern digital and print media were significantly less impactful: Television was reported by 13% of respondents, while information received via mobile phone/SMS stood at 10%. Print media, such as newspapers and magazines, were the least common source, cited by only 8.5% of the women.

Overall, these findings underscore the importance of **leveraging social gatherings and radio** as primary communication channels for FP messages. Strengthening these interpersonal and mass media platforms remains essential for expanding the reach and impact of FP information in rural communities.

These findings, coupled with the limited knowledge expressed by male participants, suggest that while overall knowledge exists, targeted efforts are needed to address knowledge gaps related to specific methods, particularly among less educated women and in areas where task delegation is implemented. Leveraging gatherings as a key information channel appears crucial for enhancing awareness of diverse contraceptive options for both women and men.

### Family planning method usage and contraceptive method preference

**Figure 3:**
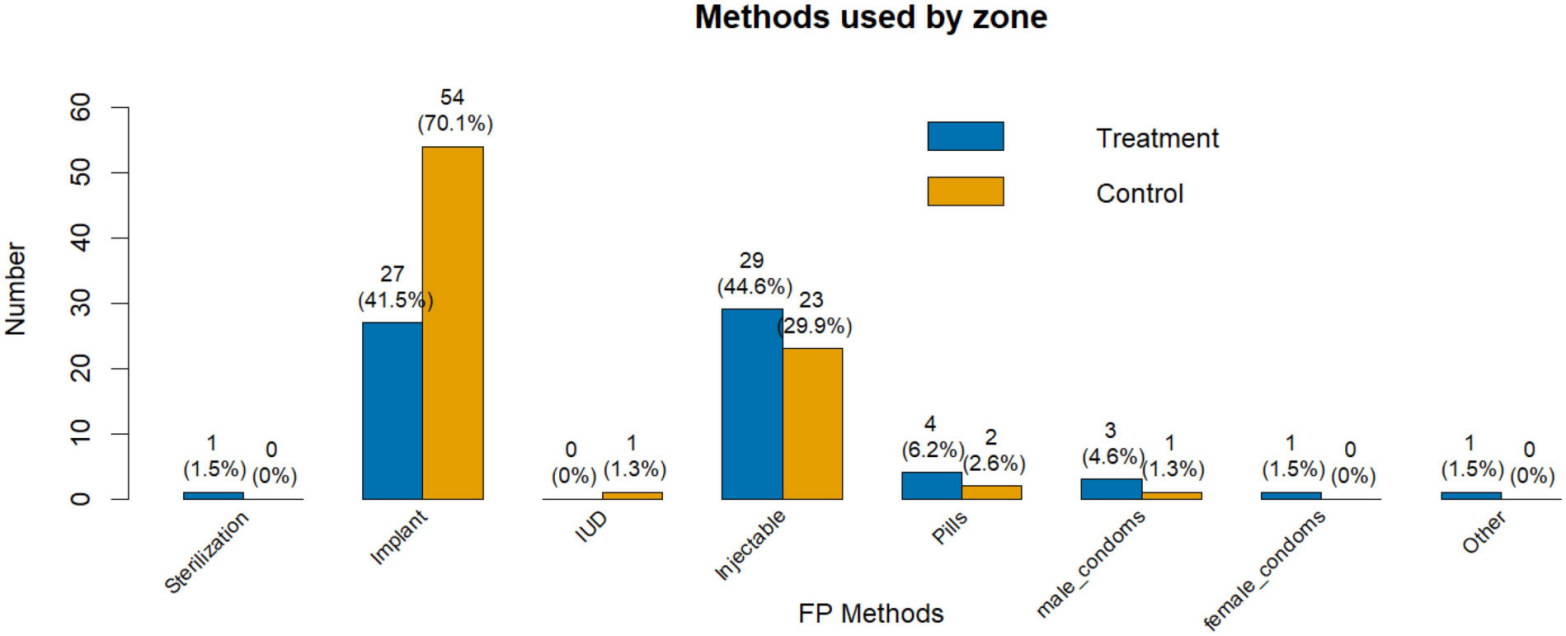
Types of family planning methods utilized by study participants.

The study revealed that 48% of women in the intervention group, where family planning tasks were delegated to ASBCs, reported using a contraceptive method. This was slightly lower than the control group, where 53% of women reported contraceptive use. Implants and injectables emerged as the most used methods across both the intervention and control groups, indicating their relative popularity and acceptance within the community (Figure 3).

### Preferred source of family planning methods

Analysis of the sources of family planning (FP) methods revealed a strong reliance on formal health facilities across both study groups. The majority of women obtained their contraceptive methods from *Centres de Santé et de Promotion Sociale* (CSPS), accounting for **82%** in the intervention group and **90%** in the control group. By contrast, only **17%** of women in the intervention group reported receiving their FP methods directly from *Agents de Santé à Base Communautaire* (ASBCs), compared to **1%** in the control group, where ASBCs primarily provided counselling or referrals.

These findings indicate that, although the intervention successfully expanded ASBCs’ roles to include the provision of certain FP methods (oral pills, DMPA-SC, and condoms), health facilities remained the dominant source of contraceptives for most women. The introduction of community-based distribution through ASBCs thus represented an additional access point rather than a substitution for facility-based services.

**Figure 4:**
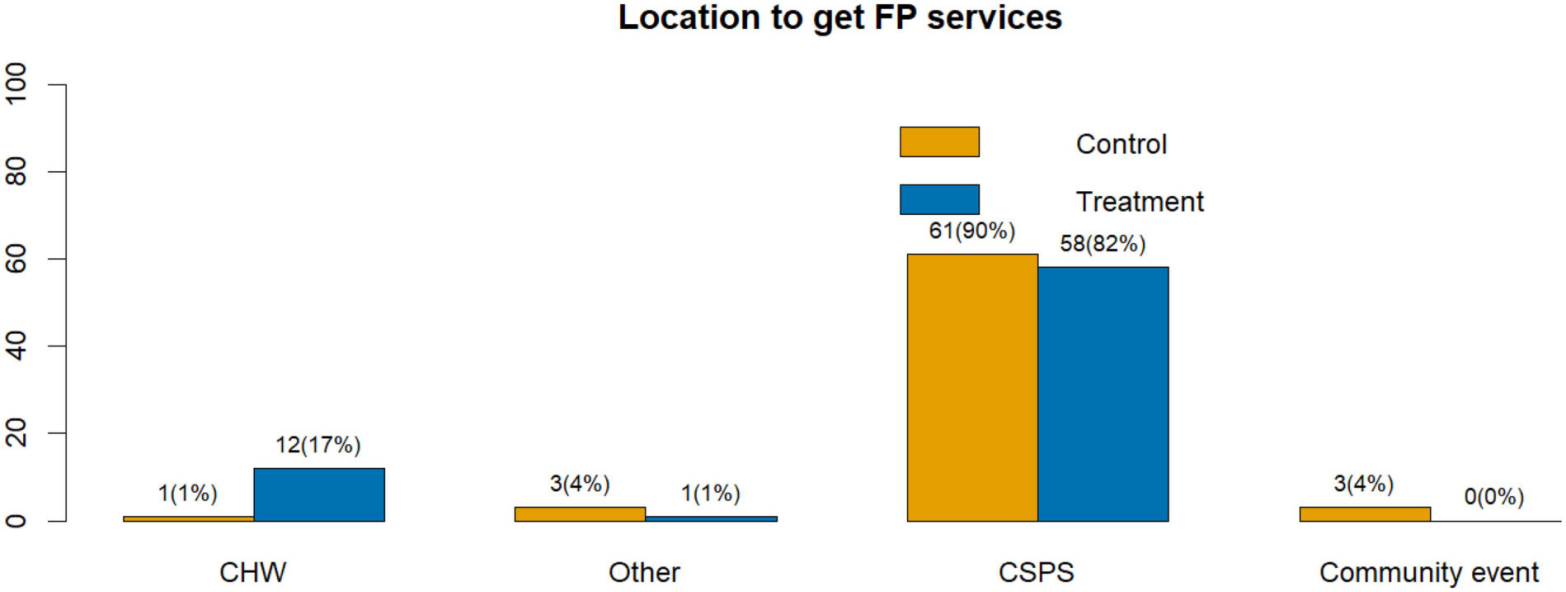
Source of modern family planning methods among study participants.

Figure 4 presents the distribution of contraceptive sources among users, illustrating the predominant role of CSPS in method provision and the comparatively limited, though emerging, contribution of ASBCs within the intervention arm.

### Factors associated with family planning use

To gain a deeper understanding of the determinants of family planning (FP) adoption, multilevel mixed-effects logistic regression analysis was employed (Table 6). The analysis identified several key factors associated with modern FP use, including the number of contraceptive methods known by a woman, marital status, and ability to discuss FP with one's partner.

**Table 6:** GLMM (logistic) results for factors associated with modern family planning use (model with partner communication).

Variables	OR^a^	95% CI^a^	*p*-value
Study area			
Control	—	—	
Treatment	0.68	0.27, 1.68	0.4
Age	1.00	0.96, 1.05	0.9
Marital status			
Single	—	—	
Married/co-habiting	21.31	2.1, 215.8	0.009
Education			
Secondary+	—	—	
Primary	1.03	0.42, 2.51	>0.9
Illiterate	0.93	0.39, 2.22	0.9
Number of contraceptive methods known	1.45	1.18, 1.79	<0.001
Discuss family planning with husband/partner			
Strongly disagree	—	—	
Strongly agree	7.57	3.44, 16.7	<0.001

a OR, odds ratio; CI, confidence interval.

Greater awareness of a wider range of contraceptive options was associated with higher odds of FP use (*p* < 0.001). Being married or cohabiting with a partner was also strongly associated with contraceptive use; women in union had approximately 21 times higher odds of using a contraceptive method compared to single women (OR = 21.3, 95% CI: 2.1–215.9, *p* = 0.009). The extremely wide confidence interval reflects the small number of single women in the sample, which leads to instability in the estimate. To improve interpretability beyond the unstable odds ratio, we additionally derived marginal predicted probabilities from the adjusted model. The adjusted predicted probability of modern contraceptive use was **41%** (95% CI: 30%–54%) among married/cohabiting women compared with **3%** (95% CI: 0%–26%) among single women, corresponding to an **absolute difference of 38 percentage points**. These predicted probabilities provide a clearer and more interpretable representation of the association than the OR alone. The detailed results are presented in Appendix Table A5.

The ability to discuss FP with one's partner was strongly associated with contraceptive use (OR = 7.57, *p* < 0.001). Although initially considered a potential mediator, its inclusion in the model did not alter the estimated effect of the intervention (Model with partner communication: OR = 0.68; Model without: OR = 0.96). The detailed estimates for the model excluding partner communication are provided in Appendix Table A6, confirming that partner communication was not on the causal pathway. Sensitivity analyses confirmed the robustness of the associations for FP knowledge and marital status.

The intervention itself did not have a statistically significant effect on modern contraceptive use. In the fully adjusted model, the intervention was associated with OR = 0.68 (95% CI: 0.27–1.68, *p* = 0.4), indicating no measurable improvement in FP uptake.

The mixed-effects logistic model showed substantial clustering at the village level, with an intraclass correlation coefficient (ICC) of 0.19, corresponding to a design effect of 2.59. This indicates that nearly one-fifth of the total variance in contraceptive use was attributable to differences between villages, justifying the use of multilevel modeling. No commune-level covariates were included, as the Directed Acyclic Graph (DAG) identified only individual-level confounders as minimally sufficient for adjustment. Random slopes were tested but did not improve model fit and were therefore not retained.

Overall, while individual-level knowledge, marital status, and partner communication were key determinants of contraceptive use, **the task-shifting intervention implemented through ASBCs did not yield a detectable effect** after accounting for clustering and confounding.

### Sociocultural barriers and stakeholders’ perceptions of family planning

The mixed-methods findings indicate that sociocultural norms and gender relations strongly shape FP behaviors. The strong association between partner discussion and FP use (OR = 7.57, *p* < 0.001) suggests that spousal communication is a key enabling factor. The large odds ratio for marital status (OR = 21.3) highlights the particular barriers faced by unmarried women, for whom FP use may be socially discouraged or stigmatized.

These findings collectively point to the importance of social and relational dynamics in shaping FP outcomes and suggest that behavioral and cultural factors may influence the effectiveness of community-based delivery strategies.

### Men’s perspectives

There were several factors that influenced men's attitudes towards contraception, while some acknowledge its potential benefits, such as facilitating improved childcare and better birth spacing, they also harbor concerns about possible adverse health effects for women, including illnesses and sterility, and the potential financial burden associated with managing complications. Furthermore, religious and traditional beliefs play a crucial role in shaping their views. Some men express that family planning practices conflicted with religious principles.

“We are Muslims, FP is forbidden because on earth we have à lot of freedom” FGD-men

“In our tradition we do not practice that” FGD-men

Also, one male participant conveyed his apprehension about contraceptive methods, stating that “*For me contraceptive methods are good but not totally, it is not good because the implants used by women move in the body. You can place it and then come to look for it and not find it and that can make you sterile.”*

### Women’s perspectives

Women's perceptions of contraceptive methods demonstrate variability, particularly when comparing the intervention and control groups. As shown in Table 7, in the intervention group, 8.1% of women strongly agree that using contraceptives will make it difficult for them to conceive in the future, whereas this figure rises to 29.5% in the control group. Additionally, while 4.4% of women in the intervention group express the belief that contraception could lead to abnormal births, this concern is held by 10% of women in the control group. Overall, these findings suggest that women residing in areas where task delegation for family planning services is implemented tend to exhibit a more favorable perception of contraceptive use.

**Table 7:** Women's perceptions of contraceptive methods by study group.

Variables	Overall *n* = 282^a^	Control *n* = 146^a^	Treatment *n* = 136^a^	*p*-value
Difficult to get pregnant after				<0.001 (Chi^2^)
Don't know/ No response	45 (16%)	25 (17.1%)	20 (14.7%)	
Totally disagree	183 (64.9%)	78 (53.4%)	105 (77.2%)	
Strongly agree	54 (19.1%)	43 (29.5%)	11 (8.1%)	
Opportunities for problems within the couple				0.007 (Chi^2^)
Don't know/ No response	49 (17.4%)	25 (17.1%)	24 (17.6%)	
Totally disagree	174 (61.7%)	81 (55.5%)	93 (68.4%)	
Strongly agree	59 (20.9%)	40 (27.4%)	19 (14%)	
Problems within the couple				0.002 (Chi^2^)
Don't know/ No response	45 (16%)	24 (16.4%)	21 (15.4%)	
Totally disagree	182 (64.5%)	83 (56.8%)	99 (72.8%)	
Strongly agree	55 (19.5%)	39 (26.7%)	16 (11.8%)	
Having abnormal children at birth				0.049 (Chi^2^)
Don't know/No response	42 (14.9%)	29 (20%)	13 (9.6%)	
Totally disagree	219 (77.7%)	102 (70%)	117 (86%)	
Strongly agree	21 (7.4%)	15 (10%)	6 (4.4%)	

a *n* (%).

### ASBCs’ perspectives

ASBCs are strong proponents of family planning, emphasizing its numerous advantages, including enabling optimal pregnancy spacing, preventing unintended pregnancies, and promoting both maternal and child health, as well as women's empowerment. ASBCs generally dismiss sociocultural and religious barriers, asserting that acceptance exists even within the Muslim community, and they highlight the historical presence of traditional contraceptive methods. An ASBC articulated this view, saying that “family planning allows the couple to have the child when they want, [and that] also it does not make women sterile”. However, it is important to note that some ASBCs do acknowledge potential side effects associated with contraceptive use, such as disturbances in the menstrual cycle.

In addition to their support for family planning, ASBCs emphasize the importance of ongoing training and supervision. One ASBC noted that despite having received initial training, periodic refreshers are crucial for maintaining high-quality service, allowing them to confidently explain contraceptive options even without reference materials. Furthermore, they appreciate the consistent supply of contraceptive products, stating that they rarely face stock shortages thanks to reliable restocking from health facilities and support from Living Goods supervisors, ensuring they can meet community needs effectively.

### Implementation challenges

Health facility managers report encountering various challenges in the implementation of FP services, primarily stemming from community reluctance and prevailing sociocultural barriers. A key obstacle identified is the pronounced preference among women for consulting female ASBCs. This preference is supported by quantitative data presented in Table 8, which demonstrates that 61% of women prefer consulting female ASBCs, compared to only 33.3% who express a preference for male ASBCs. The influence of social dynamics on service delivery is further illustrated by a key informant who explained that “it is difficult for a woman to confide in an ASBC man for a desire for FP given that her husband and the ASBC are in the same community and sometimes they are even friends [adding that] that is a difficulty”.

**Table 8:** Women's sociocultural behaviors and preferences regarding family planning by study group.

Variables	Overall *n* = 282^a^	Control *n* = 146^a^	Treatment *n* = 136^a^	*p*-value
Shyness				<0.001 (Chi^2^)
Agree	127 (45%)	83 (56.8%)	44 (32.3%)	
Disagree	135 (47.9%)	53 (36.3%)	82 (60.3%)	
No response	20 (7.1%)	10 (6.9%)	10 (7.4%)	
Go to ASBC women				0.9 (Chi^2^)
Agree	172 (61%)	87 (59.6%)	85 (62.5%)	
Disagree	88 (31.2%)	43 (29.5%)	45 (33.1%)	
No response	22 (7.8%)	16 (10.9%)	6 (4.4%)	
Go to ASBC man				0.4 (Chi^2^)
Agree	94 (33.3%)	52 (35.6%)	42 (30.9%)	
Disagree	168 (59.6%)	82 (56.2%)	86 (63.2%)	
No response	20 (7.1%)	12 (8.2%)	8 (5.9%)	

a *n* (%).

### Men’s involvement in the use of contraceptive methods and decision-making

The study reveals that there is a large majority (89%). of women using FP had informed their partners, indicating a general awareness among men. Furthermore, a considerable proportion of women (87%) reported that they consulted their partners before initiating contraceptive use.

### Partner involvement in family planning decisions

**Figure 5:**
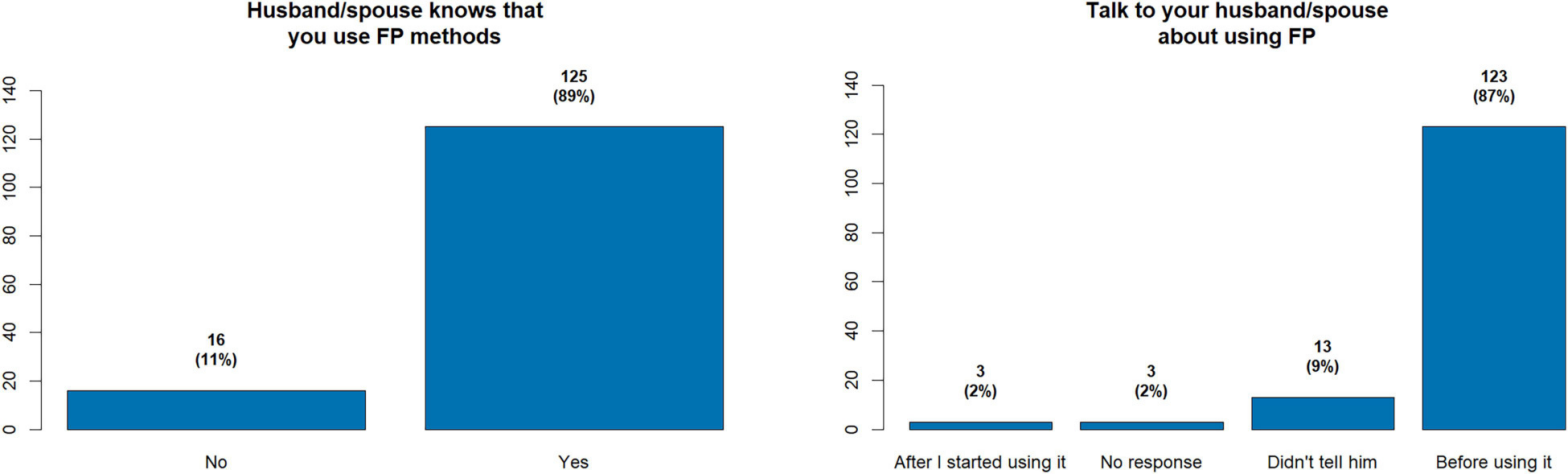
Proportion of women whose husband/spouse knows they use family planning methods, and proportion of women who discussed family planning with their husband/spouse before using contraceptives.

The data analysis, presented in the study and illustrated in Figure 5, shows varying levels of partner engagement. As detailed in Table 9, in the intervention group, where task delegation in FP was implemented, 72.1% of women reported they could negotiate with their husbands about the timing of future pregnancies, compared to 57.5% of women in the control group. Additionally, 78.7% of women in the intervention group stated they could discuss FP with their husbands, whereas only 63% of women in the control group reported the same. While these figures collectively suggest a higher degree of male involvement in FP decisions within the intervention group, causation cannot be inferred due to the cross-sectional nature of the data.

**Table 9:** Comparison of family planning decision-making and discussions between treatment and control groups.

Variables	Overall *n* = 282^a^	Control *n* = 146^a^	Treatment *n* = 136^a^	*p*-value
Change family planning method when I want				0.005 (Chi^2^)
Don't know/No response	77 (27.3%)	37 (25.3%)	40 (29.4%)	
Strongly disagree	23 (8.2%)	19 (13%)	4 (2.9%)	
Strongly agree	182 (64.5%)	90 (61.7%)	92 (67.7%)	
Discuss FP with husband/partner				0.042 (Chi^2^)
Don't know/No response	45 (15.9%)	29 (19.9%)	16 (11.7%)	
Strongly disagree	38 (13.5%)	25 (17.1%)	13 (9.6%)	
Strongly agree	199 (70.6%)	92 (63%)	107 (78.7%)	
I can decide when to have children				<0.001 (Chi^2^)
Don't know/No response	40 (14.2%)	33 (22.6%)	7 (5.1%)	
Strongly disagree	81 (28.7%)	20 (13.7%)	61 (44.9%)	
Strongly agree	161 (57.1%)	93 (63.7%)	68 (50%)	
I can negotiate with my husband when to stop having children				0.13 (Chi^2^)
Don't know/No response	55 (19.5%)	35 (24%)	20 (14.7%)	
Strongly disagree	45 (16%)	27 (18.5%)	18 (13.2%)	
Strongly agree	182 (64.5%)	84 (57.5%)	98 (72.1%)	

a *n* (%).

### Men’s attendance at family planning facilities

Contrary to the perception on spousal communication on family planning, focus group discussions highlighted that most men do not accompany their wives to health facilities when choosing a contraceptive method. This observation is supported by accounts from some women who reported that they do not inform their partners about their decision to adopt FP methods; instead, they directly visit the facility to obtain the method. For example, some men expressed that “*I have never accompanied my wife and I will never accompany her”*. In discussions about improving communication, some men suggested *that “The woman should normally inform you before using contraceptive methods, but if she does not inform you, how will you know to support her?”*. It was also noted by several men *that “Some inform the husband but on the other hand some women do not inform, however logic would dictate that you discuss to make a decision”.* This reflects the varied approaches to communication within couples regarding family planning, highlighting the influence of individual and cultural differences in decision-making.

### Implementation and effectiveness of the delegation of family planning tasks to ASBCs

#### ASBC activities and impact

The study assessed the reach and diverse activities of ASBCs within the community. It revealed that a significant proportion (55%) of women reported having received a visit from an ASBC to discuss FP in the last 12 months preceding the survey. This indicates that ASBCs are actively engaging with women in the community to promote family planning awareness and services. Among the women who reported receiving an ASBC visit, a substantial proportion (95%) were currently using a contraceptive method. This observation suggests a potential positive link between ASBC engagement and contraceptive use within the community. Statistical analysis confirmed that there was a statistically significant relationship between women receiving a visit from an ASBC and their use of contraceptive methods (*p*-value < 0.001).

The study also details the range of activities undertaken by ASBCs in their role of promoting family planning and overall health within the community. These activities include:

- Distributing contraceptive methods: ASBCs provide access to essential contraceptives, including injectables, pills, and condoms, directly within the community, increasing convenience and access.
- Providing counseling: ASBCs offer valuable guidance, information, and support to women, addressing their questions, concerns, and misconceptions about family planning.
- Conducting home visits: ASBCs proactively reach out to women in their homes, providing personalized information, education, and services, particularly to those who may face barriers in accessing health facilities.
- Organizing educational talks: ASBCs conduct community-based sessions to raise awareness about family planning, reproductive health, and related health topics, promoting knowledge and informed decision-making.

ASBCs further described their approach to providing family planning services, highlighting that there is generally no rigid, standardized protocol in place. Instead, they rely on open communication and client-centered interactions with women, focusing on discussing the advantages and disadvantages of different contraceptive methods, and conducting basic health checks before administering contraceptives. This flexible and individualized approach allows ASBCs to tailor their services to the specific needs, preferences, and health status of each woman.

Beyond family planning, ASBCs also engage in other important health-related activities within the community, such as raising awareness about the benefits of breastfeeding for maternal and child health and conducting weigh-ins to monitor child growth and nutritional status. ASBCs themselves provided descriptions of their activities, offering firsthand accounts of their community engagement and the breadth of their work. For example, ASBCs reported engaging in “*Awareness on FP, the benefits of weighing and breastfeeding, the benefits of bringing children to the hospital for care in case of illness and that for FP, we do awareness raising, home visits, maintenance, counseling and distribution of FP products and we also talk with women, we do educational talks, counseling, we inject them with the product and also have the pills”*. ASBC

Health facility in charges also provided valuable insights into the roles played by ASBCs within the community, emphasizing their contributions to community mobilization, health promotion, and referral services. Facility heads reported that “*The role they play is really mobilization, monitoring and awareness raising, they also refer cases”*. HF in charge. They also generally expressed a positive perception of the overall impact of ASBC involvement in family planning service delivery within their communities. They noted significant improvements in community awareness about family planning and increased acceptance of contraceptive methods among women.

“The impact is that women are becoming more and more awakened. Women are increasingly adhering to family planning” HF in charge

“There was really a positive impact because the ladies who were hiding to come, once they trust the ASBC, it’s over, everything is managed there and when we look at their report, we see that there is real data, and it is satisfactory” HF in charge

“In our establishment we have noticed that in the past that those who were not oriented or the rate of use which was low in some villages have been increased. Because quite simply the ASBCs lead discussion sessions and orient or motivate many women to come, even those who are reluctant. This is the far result of these services in terms of family planning.” HF in charge.

Overall, the findings suggest that ASBCs contributed to raising awareness and providing counseling and services, yet persistent sociocultural barriers, gender norms, and preferences for facility-based care limited their overall independent effect on FP use.

### Integration of quantitative and qualitative findings

To provide a comprehensive understanding of the factors shaping family planning knowledge and uptake, we developed a joint display linking quantitative predictors with illustrative qualitative quotes. This integration highlights how statistical associations observed in the survey are reinforced, nuanced, or challenged by perspectives from men, ASBCs, and health facility managers. As shown in Table 10, the joint display synthesizes these complementary insights to offer a richer interpretation of family planning behavior, knowledge, and use.

**Table 10:** Joint display of quantitative and qualitative findings on family planning behavior, knowledge and use.

Quantitative Findings	Qualitative Exemplar Quotes	Integration / Interpretation
**Knowledge of FP methods:** Higher knowledge among educated women; participation in FP talks linked to knowing 17% more methods	“ASBCs hold community talks and guide women, even those who were initially reluctant, to seek information.” (KII health facility manager)	Quantitative evidence that education and participation in FP gatherings increase knowledge is supported by qualitative reports showing that community dialogues and sensitization are key channels for disseminating information.
**Partner communication:** Women able to discuss FP with partner had much higher odds of use (OR=7.57)	“It is often the husbands who decide; if the husband is not in agreement, the woman cannot seek FP.” (FGD men) “In my opinion, the man and the woman must first reach mutual agreement before adopting family planning.” (FGD men)	Quantitative results highlight partner communication as a strong predictor. Qualitative insights reveal that decision-making is often male dominated, suggesting that interventions must directly address couple dynamics.
**Task-shifting to ASBCs**: No significant independent effect on FP use, despite 55% of intervention women receiving ASBC visits	“Many women are interested but prefer to go to the health center rather than obtain contraceptives from us.” (FGD ASBC)	Although ASBCs improved awareness, cultural and gender norms limited their impact. Preferences for facility-based services help explain why quantitative analysis did not show a significant effect.
**Preference for female providers:** 61% prefer female ASBCs	“Women rarely confide in male ASBCs; they prefer female ones.” (KII health facility manager) “Male ASBCs have difficulty reaching women… It is the female ASBCs who succeed in supporting most women who need FP.” (KII health facility manager)	A marked preference for female providers reflects gender norms and limits male ASBCs’ effectiveness. Recruiting more female ASBCs may improve service reach.
**Problems within the couple:** 20.9% report potential conflict	“…managing partner conflicts is part of the challenge, because FP often creates disputes within families.” (KII health facility manager) “If it’s for other matters I don’t mind, but if my wife consults an ASBC for contraceptives, she will change home.” (FGD men)	Conjugal tensions, rooted in male authority and mistrust, act as barriers. Both datasets highlight the need to involve men in interventions to reduce conflict and foster acceptance.
**Difficult to get pregnant after FP:** 19.1% believed contraceptives could hinder future pregnancy	“Implants move inside the body; you may not find them again and that can make women sterile” (FGD men)	Fear of infertility, fueled by misinformation, discourages uptake. This underscores the need for targeted communication strategies to dispel myths.

## Discussion

The primary objective of this study was to assess the impact of ASBC task-shifting on modern contraceptive prevalence and to identify factors associated with its use in Ziniaré. The core finding is that despite the establishment of a community-based supply chain through enhanced ASBCs, the difference in contraceptive prevalence between the intervention (48%) and control (53%) groups was not statistically significant (OR = 0.68, 95% CI: 0.27–1.68, *p* = 0.4). This result underscores that merely increasing the availability of contraceptives in the community is insufficient; success hinges on overcoming deep-seated social and behavioral barriers, which are rarely addressed within the short timeframe of an 8-month intervention. While the intervention appears to have enhanced women's awareness and engagement with family planning services, these intermediate gains did not translate into a measurable increase in modern contraceptive use within the study period.

### Socio-demographic context

Low literacy levels remain a key barrier to FP adoption in Burkina Faso, limiting women's ability to access, interpret, and act upon reproductive health information. Illiteracy was lower among women in the intervention group (45.6%) compared to the control group (60.3%), which may reflect slightly higher baseline health literacy or improved understanding through community-based counseling in local languages. This aligns with previous evidence showing that community-delivered information can enhance FP knowledge and uptake in low-literacy settings (11). Nearly all participants were married or cohabiting, reflecting entrenched cultural norms of early marriage and underscoring the critical role of male partners in FP decision-making. Given that Burkina Faso's fertility rate remains among the highest globally (5.4 children per woman) (12), community-based programs that engage couples and normalize FP discussions are essential.

### Sources of information and service access

Women in both study groups primarily reported receiving FP information through **radio** and **social gatherings**, reflecting the dominant channels for health communication in rural Burkina Faso. Radio's wide accessibility and low cost make it a key source, while social gatherings leverage community norms and peer influence to disseminate information, consistent with evidence from sub-Saharan Africa (11, 13). The study found minimal reliance on other sources such as print media, television, or mobile/SMS messaging across both groups, highlighting that digital and text-based channels currently play a limited role in this setting. This underscores the continued importance of **broad-reach and interpersonal communication strategies** for FP programming, particularly in low-literacy populations.

### Facility preference and implications for ASBC reach

The analysis of contraceptive sources revealed a strong and persistent preference for established health facilities, underscoring their central role in contraceptive provision despite the implementation of a community-based intervention. The vast majority of women across both groups relied on Centres de Santé et de Promotion Sociale (CSPS) as their primary source, 82% among users in the intervention arm and 90% in the control arm, as expected given the absence of community-based supply options in the latter. Only 17% of users in the intervention group reported obtaining their contraceptive methods directly from ASBCs.

This pattern suggests that, while ASBCs play an important role in counselling, education, and demand generation, they are not yet the main service providers for most women. The intervention effectively activated ASBCs as a new access point within the community (17% vs. 1% in control), but the overall low utilization of this channel highlights that enhanced service availability did not necessarily translate into increased community uptake.

Several factors help explain this limited reach. One of the most salient is the perception of health facilities as more confidential and trustworthy environments for obtaining FP services. In the sociocultural context of Burkina Faso, where male opposition to contraception remains common, many women prefer to seek FP services at facilities to avoid being seen or questioned by their husbands or community members. By contrast, obtaining methods through ASBCs, who live within the same communities, increases the risk of social scrutiny or gossip, which discourages use. This dynamic reflects broader gender and social norms that shape women's comfort with different service delivery channels. It is particularly pronounced among younger and less educated women, who report greater constraints on reproductive decision-making and stronger concerns about discretion (14, 15). Women in this study emphasized proximity, reliability, and above all, privacy and confidentiality as key factors influencing their choice of service source.

These findings underscore that while task-shifting successfully decentralized contraceptive provision and improved local access, its impact remains limited by perceptions of confidentiality and persistent gender norms. To enhance the reach of community-based providers, future interventions should integrate components that strengthen confidentiality safeguards, support gender-sensitive provider training, and address community norms that discourage women from openly engaging with ASBCs for family planning services.

### Method preference and gender dynamics

Differences in contraceptive method mix illustrate how the task-shifting intervention influenced service delivery. Women in the intervention group showed greater reliance on **injectables**, a short-acting method easily provided at the community level by ASBCs, whereas women in the control group favored **implants**, which require facility-based insertion. This pattern aligns with prior evidence indicating that short-acting methods are more readily integrated into community-based models (16).

A key observation was that **61% of women expressed a preference for female ASBCs**, highlighting the importance of **gender concordance** between provider and client for sensitive services such as family planning (17). In contexts where male ASBCs predominate, this preference may structurally limit the reach of task-shifting, particularly among women less comfortable discussing reproductive intentions with male providers.

These findings suggest several considerations for program design within the context of community-based family planning service delivery. First, the **gender composition of ASBCs** is critical for acceptability, indicating that communities with a sufficient proportion of female providers may achieve higher engagement. Second, **method suitability** influences utilization patterns, with short-acting methods being more amenable to community provision and thus more likely to be adopted when delivered by ASBCs. Finally, **male ASBC deployment** may require additional supportive strategies, such as community sensitization or targeted engagement initiatives, to optimize acceptability in contexts where prevailing gender norms constrain interactions between women and male providers.

Overall, these findings emphasize that both the **type of contraceptive method** and the **gender of the ASBC** affect uptake patterns, while practical recommendations for addressing these factors are presented in the “Programmatic Implications” section.

### Task-Shifting effectiveness and limitations

The absence of a significant difference in contraceptive prevalence between intervention and control groups (48% vs. 53%, OR = 0.68, 95% CI: 0.27–1.68, *p* = 0.4) contrasts with global systematic reviews reporting strong effects of task-shifting on family planning uptake (18, 19). This apparent discrepancy is likely explained by several important contextual factors specific to Ziniaré:

1. **Short intervention duration:** The eight-month implementation period may have been insufficient for measurable behavioral changes in contraceptive use to manifest, whereas most studies demonstrating strong task-shifting effects report follow-up periods of 12–24 months or longer.
2. **Ceiling effects:** Baseline contraceptive prevalence in the study area was already relatively high (48%–53%) compared to the national average of 32% (9). This limits the potential for additional measurable gains from community-based provision.
3. **Limited statistical power:** *post-hoc* calculations of the minimum detectable effect (MDE), accounting for the achieved sample size, cluster distribution, and plausible ICC values (Appendix Table A1), indicate that the study was underpowered to detect small differences between groups. Based on the achieved sample size and ICC, the minimum detectable effect was approximately 18.2–21.1 percentage points at 80%–90% power (Appendix Table A1), well above the observed 5-point difference. The observed 5-percentage-point difference in contraceptive prevalence falls below the MDE thresholds, suggesting that the non-significant result may plausibly reflect a Type II error rather than the absence of an intervention effect.
4. **Sociocultural barriers:** Persistent gender norms, male opposition to contraception, and social scrutiny in communities shaped women's comfort with using ASBCs for FP services. Even with increased accessibility through community-based providers, many women preferred facility-based services due to perceived confidentiality and trustworthiness, constraining the impact of the intervention.

By explicitly situating our findings within these local contextual factors, we provide a nuanced interpretation that complements the global evidence base and clarifies why task-shifting did not result in a statistically significant increase in contraceptive prevalence in this specific setting. Overall, the findings indicate that the ASBC-led model successfully strengthened awareness, communication, and service availability, foundational elements for long-term change, but measurable behavioral outcomes may require longer implementation and stronger sociocultural alignment.

### Predictive factors and partner involvement

Our multilevel regression analyses identified several correlates of modern contraceptive notably marital status, FP knowledge, and partner communication. Married or cohabiting women were significantly more likely to use modern methods (OR = 21.3, *p* = 0.009), and higher knowledge of FP methods was positively associated with use (OR = 1.45, *p* < 0.001), consistent with findings from Orjingene and Morgan (20), who emphasized education and knowledge as fundamental drivers of contraceptive adoption in community-based interventions.

Notably, women reporting discussion of FP with their partners exhibited markedly higher odds of contraceptive use (OR = 7.57, *p* < 0.001), aligning with multiple studies cited in the *International Journal of Public Health* (21) and underscoring spousal communication as one of the strongest predictors of FP uptake. This strong association also highlights the critical importance of male engagement strategies, as emphasized by the Ouagadougou Partnership framework for francophone West Africa, particularly in contexts where social norms influence reproductive decision-making.

Considering partner communication as a mediator clarifies the mechanism through which task-shifting interventions are expected to influence contraceptive behavior. By increasing access to FP services through ASBCs, the intervention may have enhanced opportunities for partner dialogue and engagement. Such communication is known to facilitate contraceptive adoption, although this pathway did not translate into a measurable difference in overall contraceptive use in this study. These findings emphasize that task-shifting strategies may be most effective when coupled with interventions promoting spousal communication and male engagement, rather than focusing solely on service delivery, to maximize behavioral impact.

### Programmatic implications

The evaluation of community-based family planning (FP) services in the Ziniaré district, Burkina Faso, highlights both encouraging progress and critical limitations that inform future program adaptation. Moving from high awareness to sustained uptake requires targeted modifications to the national task-shifting model. Based on study findings, the following programmatic recommendations are proposed:

(1) Extend the Implementation and Evaluation Horizon

The absence of a statistically significant increase in contraceptive prevalence after only eight months underscores the need for a longer intervention period. Social and behavioral change, particularly regarding gender norms and trust in community-based providers, requires time. We recommend that the Ministry of Health adopt a minimum 2–3-year implementation and evaluation horizon to allow ASBCs (Agents de Santé à Base Communautaire) to build credibility, normalize community-based FP services, and foster gradual changes in social acceptance, especially regarding male approval.

(2) Strengthen Confidentiality and Gender-Sensitive Service Provision

The persistent preference for facility-based FP services (82%–90%) and the strong inclination toward female ASBCs (61%) indicate that privacy and gender concordance remain decisive factors in service utilization. Two immediate programmatic adjustments are warranted:

- **Institutionalize confidentiality protocols** within ASBC training curricula, ensuring that community-level FP services are perceived as discreet and trustworthy as facility-based care.
- **Prioritize recruitment and retention of female ASBCs**, particularly in communities where cultural norms restrict male–female interactions on reproductive topics. Gender-balanced deployment should become a core principle of community health staffing.

(3) Engage Men as Partners in FP Decision-Making

Quantitative results revealed that partner communication was the strongest predictor of contraceptive use (OR = 7.57), underscoring male approval as a pivotal determinant. National task-shifting strategies should therefore integrate structured **male engagement interventions**, beyond basic awareness-raising, to address persistent myths and misconceptions (e.g., fears of sterility or implant migration) and to promote joint decision-making. Male sensitization initiatives can reposition men as supportive allies in FP uptake rather than as passive gatekeepers.

(4) Optimize Task-Shifting Operations and Supply Chain Reliability

For task-shifting to achieve its potential, operational bottlenecks must be addressed. The study identified weaknesses in ASBC training, supervision, and contraceptive supply continuity. These gaps undermine community trust and service consistency. A **robust logistics and supervision system** should therefore be institutionalized to guarantee uninterrupted contraceptive availability, ongoing ASBC capacity building, and effective integration between community and facility-based services.

(5) Strengthen Multi-Sectoral Coordination and Community Engagement

Sustained success will require close collaboration among ASBCs, health facilities, local authorities, and community leaders. Coordination mechanisms should ensure smooth referral pathways, shared accountability, and community-led feedback systems. Additionally, leveraging **existing community gatherings**, which were shown to significantly increase FP awareness, can amplify social support for contraceptive use and facilitate norm change.

### Strengths, limitations, and future research

The strength of this study lies in its mixed-methods approach, which provided the qualitative data necessary to interpret the null quantitative finding, revealing the critical, non-logistical barriers of confidentiality and male opposition, thereby generating contextually relevant, data-driven programmatic recommendations.

This study has several limitations. Its **cross-sectional and quasi-experimental design** limits causal attribution, and the study was **underpowered** to detect the observed prevalence difference. Methodologically, the exclusion of women from the qualitative interviews means that women's experiential voices are not directly represented, and the depth of narrative understanding regarding barriers, such as preferences for facility privacy, confidentiality, or social constraints, is primarily derived from provider and male perspectives. This may bias the interpretation of qualitative findings and highlights the need for future studies to include women's qualitative perspectives.

Future research should investigate the differences between women who discuss family planning (FP) with their partners and those who do not, with particular attention to the potential influence of intimate partner violence (IPV) dynamics and reproductive autonomy. Longitudinal studies are also needed to assess the sustained impact of task-shifting on FP uptake, including its scalability to other districts. Additionally, future work should explore effective strategies to enhance men's knowledge, engagement, and shared decision-making in family planning.

## Conclusion

This study demonstrates that task-shifting family planning (FP) services to community health workers (ASBCs) enhances access, awareness, and engagement at the community level in Ziniare, Burkina Faso. Women in intervention areas reported improved perceptions of FP and greater communication with partners, highlighting the role of ASBCs in fostering informed reproductive decision-making. However, persistent sociocultural and religious barriers, gender norms, and preferences for facility-based services limit the full potential of community-based interventions.

Multilevel analyses revealed that marital status, FP knowledge, and partner communication, particularly as a mediator, are critical predictors of contraceptive uptake, underscoring the importance of integrating educational and couple-centered strategies into FP programs. The findings also highlight the need for gender-sensitive approaches, continuous supervision, performance management, and reliable supply chains to support ASBCs effectively.

Overall, this study emphasizes that while task-shifting is a promising strategy to expand FP access, its success depends on addressing sociocultural constraints, promoting male engagement, and embedding ASBC activities within broader health system structures. A multi-level, contextually grounded, and performance-led approach is essential to achieving sustainable improvements in contraceptive uptake, reproductive autonomy, and equitable health outcomes.

## Data Availability

The raw data supporting the conclusions of this article will be made available by the authors, without undue reservation.
