# Supplementary material for: Task-shifting through community health workers: factors influencing access and utilization of modern family planning methods in Ziniaré, Burkina Faso
Source: Front Reprod Health. 2026 Feb 17;8:1650177. doi: 10.3389/frph.2026.1650177 (PMC12953070; doi:10.3389/frph.2026.1650177)
Supplement: Supplementary file 1 [file Datasheet1.pdf]

**Appendix 1**

Appendix Table A1. A priori sample size parameters and post-hoc minimum detectable effect (MDE) calculations based on achieved sample and cluster structure

| **Parameter** | **A priori assumptions (used in original sample size calculation)** | **Achieved study values (used in post-hoc MDE calculations)** |
|---|---|---|
| Alpha (two-sided) | 0.05 | 0.05 |
| Power | 80% | 80% and 90% |
| Baseline prevalence (p) | 0.32 | Observed pooled prevalence = 0.506 |
| Target detectable difference | 18 percentage points | — (MDE computed post-hoc) |
| Number of clusters | Not pre-specified | 27 total (13 intervention, 14 control) |
| Observed sample sizes | Required total n = 252 | $n_1$ = 136 (intervention), $n_2$ = 146 (control), total = 282 |
| Mean cluster size (m) | — | 9.10 |
| Cluster size range (min–max) | — | 1 – 20 |
| Standard deviation of cluster size | — | 3.53 |
| Coefficient of variation (CV) | — | 0.39 |
| ICC | 0.02 | 0.02 (plausible estimate) |
| Design effect (DE) | Derived from ICC | 1.19 (adjusted for unequal cluster sizes) |
| Effective sample sizes | — | $n_1$,eff = 114.4; $n_2$,eff = 122.8 |
| Post-hoc MDE (80% power) | — | ≈ 18.2 percentage points |
| Post-hoc MDE (90% power) | — | ≈ 21.1 percentage points |
| Observed group difference | — | 5 percentage points (48% vs 53%) |

Notes:

1. Post-hoc minimum detectable effects (MDEs) were calculated using the actual number of clusters (13 intervention, 14 control), the observed cluster sizes (range 1–20; mean 9.10; SD 3.53; CV 0.39), and a plausible ICC of 0.02. Calculations applied the standard design-effect–adjusted formula for two-sample comparisons of proportions.
2. The resulting MDEs at 80% and 90% power (≈18.2 and ≈21.1 percentage points, respectively) exceed the observed difference of 5 percentage points between groups. This indicates that the study was underpowered to detect the small effect observed, and that the non-significant result may plausibly reflect a Type II error rather than the absence of an intervention effect.

Appendix Table A2. Variation in Adjusted DE, Effective Sample Sizes, and MDE at 80% and 90% Power Across ICC Values

| **ICC** | **ajusted DE** | **$n_1$,eff** | **$n_2$,eff** | **MDE 80%** | **MDE 90%** |
|---|---|---|---|---|---|
| 0.01 | 1.095 | 124.2 | 133.3 | 17.4% | 20.2% |
| 0.02 | 1.190 | 114.3 | 122.7 | 18.2% | 21.1% |
| 0.03 | 1.284 | 105.9 | 113.7 | 18.9% | 21.9% |

Notes: The table shows that MDEs remain substantially higher than the observed difference of 5 percentage points, even when varying ICC from 0.01 to 0.03. This confirms that the study was underpowered to detect the small effect observed, and that the non-significant result may plausibly reflect a Type II error rather than the absence of an intervention effect.

Appendix Table A3. Commune-Level Number of ASBCs and Population Estimates

| **Commune** | **Number of ASBCs** | **Women of Reproductive Age Population per ASBC (approx.)** |
|---|---|---|
| Nagreongo | 40 | 221 |
| Loumbila | 62 | 158 |
| Dapelogo | 60 | 198 |
| Ourgou Manéga | 60 | 108 |
| Absouya | 38 | 249 |
| Ziniaré | 102 | 143 |
| Zitenga | 112 | 121 |

Sources: 2019 national census (RGPH)

Appendix Table A4. Model Diagnostics, ICCs, Design Effects, and Goodness-of-Fit Indices

| Outcome | Model | Link | Random effects | ICC commune | ICC village | DEFF commune | DEFF village | Dispersion (DHARMa) | p-value | Sigma | Pearson $\chi^2$/df | AIC | AICc | BIC | $R^2$ (marg.) | $R^2$ (cond.) | RMSE | Log-loss |
|---|---|---|---|---|---|---|---|---|---|---|---|---|---|---|---|---|---|---|
| *Contraceptive methods known* | COM-Poisson GLMM | Log | Random intercepts: commune/ village | 0.02 | 0.025 | 1.77 | 1.2 | 0.859 | 0.354 | 0.408 | – | 1077.1 | 1078 | 1113.5 | 0.024 | 0.082 | 1.376 | – |
| *Modern family planning use* | Logistic GLMM | Logit | Random intercept: village | – | 0.19 | – | 2.59 | 1.035 | 0.586 | 1 (fixed in logit) | 0.8 | 327.5 | 328 | 367.5 | 0.438 | 0.548 | 0.397 | 0.474 |

Appendix Table A5. Adjusted predicted probabilities of modern contraceptive use by marital status

| **Marital status** | **Predicted probability (%)** | **95% Confidence Interval (%)** | **Absolute difference vs single (%)** |
|---|---|---|---|
| Single | 3 | 0 – 26 | – |
| Married / cohabiting | 41 | 30 – 54 | 38 |

Notes:

- Predicted probabilities derived from the fully adjusted multilevel logistic regression model (adjusting for age, education, number of contraceptive methods known, partner communication, including a random intercept for village).
- Absolute difference calculated as Married/cohabiting – Single.
- OR for marital status is reported in Table 6 of the main manuscript, but predicted probabilities provide a more interpretable measure of effect magnitude.

Appendix Table A6. GLMM (logistic) results for factors associated with modern family planning use (Model with partner communication)

| **Variables** | **OR** | **95% CI** | **p-value** |
|---|---|---|---|
| **Study area** | | | |
| Control | — | — | |
| Treatment | 0.96 | 0.44, 2.08 | 0.9 |
| **Age** | 1.02 | 0.97, 1.07 | 0.4 |
| **Marital status** | | | |
| Single | — | — | |
| Married/co-habiting | 16.95 | 1.9, 152.4 | 0.01 |

| | | | |
|---|---|---|---|
| **Education** | | | |
| Secondary + | — | — | |
| Primary | 0.73 | 0.32, 1.66 | 0.4 |
| Illiterate | 0.68 | 0.30, 1.53 | 0.35 |
| **Number of contraceptive methods known** | 1.49 | 1.23, 1.81 | <0.001 |
| **ICC (Intraclass Correlation)** | 0.14 | | |
| **DEFF (Design Effect)** | 2.14 | | |
| OR = Odds Ratio, CI = Confidence Interval | | | |

**Appendix 2: R Session Information**

All analyses were performed using R. The version of R and the packages used are listed below to ensure reproducibility of the results.

R version 4.4.3 (2025-02-28 ucrt)

Platform: x86_64-w64-mingw32/x64

Running under: Windows 11 x64 (build 26100)

Main packages used:

glmmTMB_1.1.12

lme4_1.1-37

DHARMa_0.4.7

COMPoissonReg_0.8.1

performance_0.14.0

dplyr_1.1.4
